# Dataset on Islamic ethical work behavior among Bruneian Malay Muslim teachers with measures concerning religiosity and theory of planned behavior

**DOI:** 10.1016/j.dib.2020.105157

**Published:** 2020-01-21

**Authors:** Nur Amali Aminnuddin

**Affiliations:** Academy of Brunei Studies, Universiti Brunei Darussalam, Brunei

**Keywords:** Islam, Muslim, Work ethic, Ethical behavior, Islamic work ethic, Religiosity, Theory of planned behavior, Brunei

## Abstract

The data presents an examination of Islamic ethical work behavior of Malay Muslim teachers in Brunei through religiosity and theory of planned behavior. The total number of participants was 370 Bruneian Malay Muslim teachers. The participants were sampled from two different types of school systems being non-religious schools and religious schools, with five schools each. By documenting information of the data, this data article presented the demographic characteristics of participants, and reliability and correlation of measures involved. Analyses of the data can provide insights into determinants and in predicting Islamic ethical work behavior. Furthermore, the data will be useful for researchers and policymakers that are interested in knowing the current situation of religiosity and behavior in the country. It can also be used as references in developing interventions, promoting and facilitating Islamic ethical work behavior in the workplace.

**Table:** Specifications Table

Subject	Psychology (General); Social Psychology
Specific subject area	Psychology of Religion; Religion in the Workplace; I–O psychology; Ethical Behavior; Organizational Behavior; Society, Culture, and Religion
Type of data	Table
How data were acquired	Data was acquired through a cross-sectional survey. Printed questionnaires were distributed to targeted population.
Data format	Raw Analyzed
Parameters for data collection	Samples consisted of Malay Muslim teachers in Brunei. They were sampled from two different types of school systems: non-religious schools and religious schools.
Description of data collection	School authorities were contacted and liaise with to distribute questionnaires, which were then collected after one month.
Data source location	District: Belait, Tutong, and Brunei-Muara Country: Brunei
Data accessibility	Data is included with this article, and on Mendeley Data Repository: Mendeley Data https://data.mendeley.com/datasets/j22dcftx7y/draft?a=b90c5bf3-72c1-4060-93a9-592e339324d5
Related research article	Article 1: Aminnuddin, N. A. (2019). Predicting Islamic ethical work behavior using the theory of planned behavior and religiosity in Brunei. The Journal of Behavioral Science, 14(1), 1–13. Retrieved from https://www.tci-thaijo.org/index.php/IJBS/article/view/147140 Article 2: Aminnuddin, N. A. (2019). Demographic factors and religious dimensions as predictors of Islamic ethical work behavior in Brunei. Psychological Thought. 12(2), 185–201. https://doi.org/10.5964/psyct.v12i2.386

**Table:** 

**Value of the Data** • The data exhibits the interplay of religion and theory of planned behavior that can help to understand intention and actualization of Islamic ethical work behavior. • These data can be useful for researchers in social psychology, psychology of religion, and behavioral sciences, as well as others who are interested to have a clearer picture of religion and ethical behavior, including those in human resource management. • Considering limited availability of studies that examined determinants of Islamic ethical work behavior [1] and predicting it [2], the data paper can provide a starting point in further replicating and extending literature, especially in other countries. • Researchers can examine group differences based on demographic variables, including gender and school system. • Social desirability and attitude in the data provide a snapshot of how the behavior in question is viewed at this specific time period, which can be used as reference point. • The data represents valuable information concerning the notion that religion is integral in the Bruneian society where it permeates every aspects of life, even though religiosity level may differ depending on the dimensions, of which this information can be useful for researchers and policymakers.

## 1 Data description

The data comprised of survey data that examined Islamic ethical work behavior (see “Dataset” file under supplementary material). Data included were demographics, and measures on religiosity and theory of planned behavior. All items had been properly labeled with values assigned, including ratings. Sampled population consisted of a convenience sample of Malay Muslim teachers in Brunei (370 teachers). Some demographics information were missing. The sample was drawn from non-religious schools (218 teachers) and religious schools (152 teachers). The participants were made up of 79 males and 289 females. Majority of them had a bachelor's degree (69.89%), followed by master's degree (25.14%) and diploma (4.97%). The participants also reported that religion influenced their life: perception of personal religiosity (88.83%), upbringing (85.01%), and work environment (83.88%). Demographics are presented (see Table 1).

**Table 1:** Demographics of participants (N = 370).

Variables	Category	*N*	*M*	*SD*	%
School System	Non-religious schools	218			58.92%
	Religious schools	152			41.08%
	Total	370	1.59	0.49	
Age Group	21–25	10			2.72%
	26–30	59			16.08%
	31–35	98			26.70%
	36–40	85			23.16%
	41–50	96			26.16%
	50 and above	19			5.18%
	Total	367	3.69	1.24	
Gender	Male	79			21.47%
	Female	289			78.53%
	Total	368	1.79	0.41	
Marital status	Single	90			24.52%
	Married	277			75.48%
	Total	367	1.75	0.43	
Education level	Diploma	18			4.97%
	Bachelor's degree	253			69.89%
	Master's degree	91			25.14%
	Total	362	2.20	0.51	
Employment status	Fulltime	366	1.00	0.00	100.00%
Work experience	5 years or less	79			21.41%
	6–10 years	94			25.47%
	11–20 years	149			40.38%
	21 years or more	47			12.74%
	Total	369	2.44	0.97	
Do you consider yourself as religious?	No	2			0.54%
	Neutral	39			10.63%
	Yes	326			88.83%
	Total	367	2.88	0.34	
Do you consider yourself growing up in an environment influenced by religion?	No	6			1.63%
	Neutral	49			13.35%
	Yes	312			85.01%
	Total	367	2.83	0.41	
Do you consider your current work environment influenced by religion?	No	10			2.73%
	Neutral	49			13.39%
	Yes	307			83.88%
	Total	366	2.81	0.46	

## 2 Experimental design, materials, and methods

This data article aimed at documenting data to examine Islamic ethical work behavior, grounded on religiosity and theory of planned behavior. The data had been used for several papers [1,2] and still has research values. The parameter of sample population was for the person to be a teacher of Malay ethnicity, religion being Islam, and of Bruneian nationality. Out of Brunei's four districts, Brunei-Muara has the highest population density, followed by Belait, and then Tutong, with Temburong having the lowest. Statistics for the year 2016 of schools and teachers were taken from Brunei Darussalam Statistical Yearbook 2016 [3]. There was a total of 175 public schools with 8470 teachers in Brunei Darussalam: 103 schools with 6280 teachers in Brunei-Muara, 24 schools with 945 teachers in Belait, 36 schools with 1013 teachers in Tutong, and 12 schools with 232 teachers in Temburong.

The numbers of schools and teachers were concentrated mostly in Brunei-Muara. The numbers were roughly similar in Belait and Tutong, while the numbers were extremely and negligibly small in Temburong. Hence, Temburong is excluded from this study due to a very low population, and the target sample population from the other three districts was deemed as adequate.

Two types of school systems were selected: non-religious schools and religious schools. For non-religious schools, the following were selected: Sekolah Menengah Sayyidina Ali in Belait; Pusat Tingkatan Enam Tutong in Tutong; and Sekolah Menengah Sultan Muhammad Jamalul Alam, Sekolah Menengah Sayyidina Hasan, and Pusat Tingkatan Enam Meragang in Brunei-Muara. For religious schools, in Belait and Tutong, there was only one in each district. In Brunei-Muara, there were only four of these schools. Therefore, for religious schools, the following five schools were selected: Sekolah Arab Belait in Belait; Ma'had Islam Brunei in Tutong; and Sekolah Persediaan Arab Sungai Akar, Sekolah Ugama Arab Menengah Perempuan Raja Isteri Pengiran Anak Damit, and Sekolah Menengah Arab Lelaki Hassanal Bolkiah in Brunei-Muara.

Relevant authorities were contacted to get approval. Following approval, copies of questionnaires were sent to liaison officers at schools to be distributed to participants. Liaison officers were strictly instructed to only distribute it among local Malay Bruneians. Questionnaires were to be completed anonymously. Participants were also informed they have the choice not to return the questionnaires or to submit an uncompleted copy.

In the dataset, for theory of planned behavior, the measures were attitude toward behavior (ATB), subjective norm (SN), perceived behavioral control (PBC), and behavioral intention (BI). Islamic ethical work behavior in the dataset was labeled as IEWB and measured only with a single item. ATB has two sub-measures: instrumental (ATBI) and experiential (ATBE). SN has injunctive (SNI) and descriptive (SND). PBC has capacity (PBCC) and autonomy (PBCA). Each sub-measure had three items. Items were labeled with numbers (e.g., ATBI1 and SND3). The process of developing the measures were done adhering to several guidelines [4,5]. Items, including labels and ratings, will further be explained later on, with more comprehensive detail as supplementary material (see “Annotated Questionnaire” file under supplementary material). This is because each item has different ratings and choices.

In order to measure religiosity, seven scales from Psychological Measures of Islamic Religiosity were used [6]. The scales were Islamic beliefs scale (IBS) with 5 items, Islamic ethical principles scale (IEPS) with 10 items, Islamic universality scale (IUS) with 4 items, Islamic duty scale (IDS) with 5 items, Islamic obligation scale (IOS) with 5 items, Islamic exclusivism scale (IES) with 4 items, and global religiousness scale (GRS) with 2 items. Similar to measures of theory of planned behavior, religiosity items were also assigned with numbers (e.g., IBS1 and IEPS5). Items, including labels and ratings, will further be explained later on, with more comprehensive detail as supplementary material (see “Annotated Questionnaire” file under supplementary material). This is because in some scales, items have varying ratings and choices.

In the dataset itself, all items for both measures of theory of planned behavior and religiosity were properly labeled. Ratings were also provided in the dataset. Furthermore, items were computed to conceive their respective measure's mean values, and then labeled as their composite counterpart (e.g., all items under ATB were averaged resulting to ATBMean in the dataset).

However, due to the dataset containing multiple measures and their items having varying ratings (even within measures), an annotated questionnaire is attached for a clearer picture of the data (see “Annotated Questionnaire” file under supplementary material). The annotated questionnaire provides list of items in the questionnaires that were distributed to participants, and composite variables that had been computed, with annotations, including labels and ratings as assigned in the dataset.

Reliabilities of instruments (see Table 2) and correlations between variables (see Table 3) were also reported. Measures concerning theory of planned behavior had no items deleted. However, for religiosity, one item was removed from IES to improve reliability. For IBS, respondents had a consensus of agreeing to a single rating, which led to inability to evaluate its reliability. All showed adequate evidence for reliability, while correlation pairings were mostly significant, with the exception of pairings with IOS.

**Table 2:** Reliability of measures.

Scale	Items	Reliability	Item deleted	Final Reliability
ATB	ATBI1 to ATBI3, and ATBE1 to ATBE3	.93	–	.93
SN	SNI1 to SNI3, and SND1 to SND3	.89	–	.89
PBC	PBCC1 to PBCC3, and PBCA1 to PBCA3	.71	–	.71
BI	BI1 to BI6	.91	–	.91
IBS	IBS1 to IBS5	–	–	–
IEPS	IEPS1 to IEPS10	.91	–	.91
IUS	IUS1 to IUS4	.81	–	.81
IDS	IDS1 to IDS5	.67	–	.67
IOS	IOS1 to IOS5	.79	–	.79
IES	IES1 to IES4	.35	IES3	.77
GRS	GRS1 and GRS2	.89	–	.89

**Table 3:** Correlation of measures.

	ATB	SN	PBC	BI	RB	IEPS	IUS	IDS	IOS	IES	GRS
ATB	1	.63**	.68**	.88**	.65**	.59**	.54**	.45**	.06	.33**	.35**
SN		1	.63**	.63**	.70**	.41**	.41**	.27**	.05	.24**	.38**
PBC			1	.69**	.69**	.45**	.39**	.30**	.03	.27**	.31**
BI				1	.65**	.53**	.45**	.40**	.06	.26**	.33**
RB					1	.51**	.48**	.38**	.13	.22**	.39**
IEPS						1	.71**	.36**	.33**	.23**	.39**
IUS							1	.39**	.23**	.26**	.37**
IDS								1	.10	.23**	.36**
IOS									1	.09	.09
IES										1	.19**
GRS											1

** ≤ 0.01.
