# Supplementary material for: Dataset on Islamic ethical work behavior among Bruneian Malay Muslim teachers with measures concerning religiosity and theory of planned behavior
Source: Data Brief. 2020 Jan 21;29:105157. doi: 10.1016/j.dib.2020.105157 (PMC6997808; doi:10.1016/j.dib.2020.105157)
Supplement: Multimedia component 1 [file mmc1.docx]

# Annotated Questionnaire

This file contained an annotated version of the questionnaire. This supplementary file is structured into four sections: demographic information, theory of planned behavior, Islamic religiosity, and computed variables. The first three sections represented items that were in the distributed questionnaire to targeted sample population. All three sections were in the questionnaire. The fourth section is the computed variables in the dataset.

Firstly, the items will be presented. Following that, a box containing details of the items (if any) will be at the end of the items/measures/scales where appropriate. In the dataset, the first variable is a running number for unique identifier (UID). For some responses that had no value assigned in dataset, there is no responses counted. But ratings for items under theory of planned behavior, only the first, middle, and last ratings were assigned values in the dataset. All missing values are coded as 999.00.

# 1. Demographic Information

The following are the demographic items for data collection:

| 1. | Institusi: |  | Sekolah Arab |  | Sekolah Aliran Utama |  |
| --- | --- | --- | --- | --- | --- | --- |
|  | *Institution:* |  | *Arabic schools* |  | *Mainstream schools* |  |

| **Variable info in dataset**  Name: School_System  Label: School System  Value:  1.00 = Non-religious schools  2.00 = Religious schools  Note: Arabic schools are religious schools where it involves an Islamic religious syllabus, compared to the mainstream schools which are using conventional / non-religious syllabus. |
| --- |

| 2. | Umur: ___ Tahun |  | 21-25 |  | 36-40 |  |
| --- | --- | --- | --- | --- | --- | --- |
|  | *Age: ___ Years old* |  | 26-30 |  | 41-50 |  |
|  |  |  | 31-35 |  | 50 dan keatas / *50 and above* | |

| **Variable info in dataset**  Name: Age_Group  Label: Age Group  Value:  1.00 = 21-25  2.00 = 26-30  3.00 = 31-35  4.00 = 36-40  5.00 = 41-50  6.00 = 50 and above |
| --- |

| 3. | Jantina: |  | Lelaki |  | Perempuan |  |
| --- | --- | --- | --- | --- | --- | --- |
|  | *Gender:* |  | *Male* |  | *Female* |  |

| **Variable info in dataset**  Name: Gender  Label: Gender  Value:  1.00 = Male  2.00 = Female |
| --- |

| 4. | Agama: |  | Islam |  |  |  |
| --- | --- | --- | --- | --- | --- | --- |
|  | *Religion:* | **NOTA: Jangan teruskan kaji selidik jika bukan beragama Islam** | | | | |
|  |  | ***NOTE: Do not continue the survey if not a Muslim*** | | | |  |

| **Variable info in dataset**  Name: Religion  Label: Religion  Value:  1.00 = Islam |
| --- |

| 5. | Etnik: |  | Melayu |  | Lain-lain. Sila nyatakan: |  |
| --- | --- | --- | --- | --- | --- | --- |
|  | *Ethnicity:* |  | *Malay* |  | *Others. Please specify:* |  |

| **Variable info in dataset**  Name: Ethnicity  Label: Ethnicity  Value:  1.00 = Malay |
| --- |

| 6. | Warganegara: |  | Brunei |  | Lain-lain. Sila nyatakan: |  |
| --- | --- | --- | --- | --- | --- | --- |
|  | *Nationality:* |  | *Brunei* |  | *Others. Please specify:* |  |

| **Variable info in dataset**  Name: Nationality  Label: Nationality  Value:  1.00 = Bruneian |
| --- |

| 7. | Taraf perkahwinan: |  | Bujang / *Single* |  | Berkahwin / *Married* |  |
| --- | --- | --- | --- | --- | --- | --- |
|  | *Marital status:* |  | Cerai / *Divorced* |  | Duda / Janda / *Widowed* |  |
|  |  |  | Berasingan / *Separated* | | |  |

| **Variable info in dataset**  Name: Marital_Status  Label: Marital Status  Value:  1.00 = Single  2.00 = Married |
| --- |

| 8. | Tahap pendidikan: |  | Diploma |  | Ijazah Kedoktoran / *PhD* |
| --- | --- | --- | --- | --- | --- |
|  | *Education level:* |  | Sarjana Muda / *Bachelor's Degree* | | |
|  |  |  | Sarjana / *Master's Degree* | | |

| **Variable info in dataset**  Name: Education_Level  Label: Education Level  Value:  1.00 = Diploma  2.00 = Bachelor’s degree  3.00 = Master’s degree |
| --- |

| 9. | Status pekerjaan: |  | Jawatan tetap |  | Jawatan sementara |  |
| --- | --- | --- | --- | --- | --- | --- |
|  | *Employment status:* |  | *Full time* |  | *Part time* |  |
|  |  |  | Kontrak |  |  |  |
|  |  |  | *Contract* |  |  |  |

| **Variable info in dataset**  Name: Employment_Status  Label: Employment Status  Value:  1.00 = Full time |
| --- |

| 10. | Pengalaman kerja dalam mengajar:___ Tahun |  | 5 atau kurang |  | 11-20 |  |
| --- | --- | --- | --- | --- | --- | --- |
|  |  |  | *5 or less* |  |  |  |
|  | *Work experience in teaching:___ Years* |  | 6-10 |  | 21 atau lebih / *21 or more* | |
|  |  |  |  |  |  |  |

| **Variable info in dataset**  Name: Work_Experience  Label: Work Experience  Value:  1.00 = 5 years or less  2.00 = 6-10 years  3.00 = 11-20 years  4.00 = 21 years or more |
| --- |

| 11. | Adakah anda menganggap diri anda sebagai seorang yang berpegang pada agama? | | | | |
| --- | --- | --- | --- | --- | --- |
|  | *Do you consider yourself as religious?* | | | | |
|  |  |  | Ya / *Yes* |  | Tidak / *No* |
|  |  |  | Neutral |  |  |

| **Variable info in dataset**  Name: Islam_Religious  Label: Islam - Religious  Value:  1.00 = No  2.00 = Neutral  3.00 = Yes |
| --- |

| 12. | Adakah anda menganggap diri anda membesar dalam persekitaran yang dipengaruhi agama? | | | | | |
| --- | --- | --- | --- | --- | --- | --- |
|  | *Do you consider yourself growing up in an environment influenced by religion?* | | | | | |
|  |  |  | Ya / *Yes* |  | Tidak / *No* |  |
|  |  |  | Neutral |  |  |  |

| **Variable info in dataset**  Name: Islam_Upbringing  Label: Islam - Upbringing  Value:  1.00 = No  2.00 = Neutral  3.00 = Yes |
| --- |

| 13. | Adakah anda menganggap persekitaran kerja anda sekarang dipengaruhi oleh agama? | | | | |  |
| --- | --- | --- | --- | --- | --- | --- |
|  | *Do you consider your current work environment influenced by religion?* | | | | |  |
|  |  |  | Ya / *Yes* |  | Tidak / *No* |  |
|  |  |  | Neutral |  |  |  |

| **Variable info in dataset**  Name: Islam_Workplace  Label: Islam - Workplace  Value:  1.00 = No  2.00 = Neutral  3.00 = Yes |
| --- |

# 2. Theory of Planned Behavior

## 2.1 Attitude Toward Behavior

## 2.1.1 Attitude Toward Behavior – Injunctive (ATBI)

1. Bagi saya, berkerja dengan cara yang beretika mengikut ajaran Islam setiap hari di tempat kerja, adalah:

*For me to work in an ethical manner according to Islamic teaching every day at the workplace, would be:*

| Tidak bagus | 1 | 2 | 3 | 4 (Neutral) | 5 | 6 | 7 | Bagus |
| --- | --- | --- | --- | --- | --- | --- | --- | --- |
| *Bad* |  |  |  |  |  |  |  | *Good* |

| **Variable info in dataset**  Name: ATBI1  Label: ATBI1  Rating: 1 to 7  Value:  1.00 = Bad  4.00 = Neutral   1. Good |
| --- |

1. Bagi saya, berkerja dengan cara yang beretika mengikut ajaran Islam setiap hari di tempat kerja, adalah:

*For me to work in an ethical manner according to Islamic teaching every day at the workplace, would be:*

| Memudaratkan | 1 | 2 | 3 | 4 (Neutral) | 5 | 6 | 7 | Berfaedah |
| --- | --- | --- | --- | --- | --- | --- | --- | --- |
| *Harmful* |  |  |  |  |  |  |  | *Beneficial* |

| **Variable info in dataset**  Name: ATBI2  Label: ATBI2  Rating: 1 to 7  Value:  1.00 = Harmful  4.00 = Neutral  7.00 = Beneficial |
| --- |

1. Bagi saya, berkerja dengan cara yang beretika mengikut ajaran Islam setiap hari di tempat kerja, adalah:

*For me to work in an ethical manner according to Islamic teaching every day at the workplace, would be:*

| Tidak berbaloi | 1 | 2 | 3 | 4 (Neutral) | 5 | 6 | 7 | Berbaloi |
| --- | --- | --- | --- | --- | --- | --- | --- | --- |
| *Worthless* |  |  |  |  |  |  |  | *Useful* |

| **Variable info in dataset**  Name: ATBI3  Label: ATBI3  Rating: 1 to 7  Value:  1.00 = Worthless  4.00 = Neutral  7.00 = Useful |
| --- |

## 2.1.2 Attitude Toward Behavior – Experiential (ATBE)

1. Bagi saya, berkerja dengan cara yang beretika mengikut ajaran Islam setiap hari di tempat kerja, adalah:

*For me to work in an ethical manner according to Islamic teaching every day at the workplace, would be:*

| Tidak menyenangkan | 1 | 2 | 3 | 4 (Neutral) | 5 | 6 | 7 | Menyenangkan |
| --- | --- | --- | --- | --- | --- | --- | --- | --- |
| *Unpleasant* |  |  |  |  |  |  |  | *Pleasant* |

| **Variable info in dataset**  Name: ATBE1  Label: ATBE1  Rating: 1 to 7  Value:  1.00 = Unpleasant  4.00 = Neutral  7.00 = Pleasant |
| --- |

1. Bagi saya, berkerja dengan cara yang beretika mengikut ajaran Islam setiap hari di tempat kerja, adalah:

*For me to work in an ethical manner according to Islamic teaching every day at the workplace, would be:*

| Tidak diingini | 1 | 2 | 3 | 4 (Neutral) | 5 | 6 | 7 | Diingini |
| --- | --- | --- | --- | --- | --- | --- | --- | --- |
| *Undesirable* |  |  |  |  |  |  |  | *Desirable* |

| **Variable info in dataset**  Name: ATBE2  Label: ATBE2  Rating: 1 to 7  Value:  1.00 = Undesirable  4.00 = Neutral  7.00 = Desirable |
| --- |

1. Bagi saya, berkerja dengan cara yang beretika mengikut ajaran Islam setiap hari di tempat kerja, adalah:

*For me to work in an ethical manner according to Islamic teaching every day at the workplace, would be:*

| Membosankan | 1 | 2 | 3 | 4 (Neutral) | 5 | 6 | 7 | Menyeronokkan |
| --- | --- | --- | --- | --- | --- | --- | --- | --- |
| *Boring* |  |  |  |  |  |  |  | *Enjoyable* |

| **Variable info in dataset**  Name: ATBE3  Label: ATBE3  Rating: 1 to 7  Value:  1.00 = Boring  4.00 = Neutral  7.00 = Enjoyable |
| --- |

## 2.2 Subjective Norm

## 2.2.1 Subjective Norm – Injunctive (SNI)

1. Kebanyakan orang yang penting pada saya _____ saya berkerja secara beretika mengikut ajaran Islam setiap hari di tempat kerja.

*Most people who are important to me _____ of me working in an ethical manner according to Islamic teaching every day at the workplace.*

| Tidak menerima | 1 | 2 | 3 | 4 (Neutral) | 5 | 6 | 7 | Menerima |
| --- | --- | --- | --- | --- | --- | --- | --- | --- |
| *Disapprove* |  |  |  |  |  |  |  | *Approva* |

| **Variable info in dataset**  Name: SNI1  Label: SNI1  Rating: 1 to 7  Value:  1.00 = Disapprove  4.00 = Neutral  7.00 = Approve |
| --- |

1. Kebanyakan orang dalam hidup saya, _____ saya berkerja secara beretika mengikut ajaran Islam setiap hari di tempat kerja.

*Most people in my life _____ me to work in an ethical manner according to Islamic teaching every day at the workplace.*

| Tidak menggalakkan | 1 | 2 | 3 | 4 (Neutral) | 5 | 6 | 7 | Menggalakkan |
| --- | --- | --- | --- | --- | --- | --- | --- | --- |
| *Discourage* |  |  |  |  |  |  |  | *Encourage* |

| **Variable info in dataset**  Name: SNI2  Label: SNI2  Rating: 1 to 7  Value:  1.00 = Discourage  4.00 = Neutral  7.00 = Encourage |
| --- |

1. Kebanyakan rakan sekerja saya, menjangka saya akan berkerja secara beretika mengikut ajaran Islam, setiap hari di tempat kerja.

*Most of my colleagues expect me to work in an ethical manner according to Islamic teaching every day at the workplace.*

| Salah | 1 | 2 | 3 | 4 (Neutral) | 5 | 6 | 7 | Betul |
| --- | --- | --- | --- | --- | --- | --- | --- | --- |
| *False* |  |  |  |  |  |  |  | *True* |

| **Variable info in dataset**  Name: SNI3  Label: SNI3  Rating: 1 to 7  Value:  1.00 = False  4.00 = Neutral  7.00 = True |
| --- |

## 2.2.2 Subjective Norm – Descriptive (SND)

1. Kebanyakan orang yang penting pada saya, berkerja secara beretika mengikut ajaran Islam setiap hari di tempat kerja.

*Most people who are important to me work in an ethical manner according to Islamic teaching every day at the workplace.*

| Tidak mungkin | 1 | 2 | 3 | 4 (Neutral) | 5 | 6 | 7 | Mungkin |
| --- | --- | --- | --- | --- | --- | --- | --- | --- |
| *Unlikely* |  |  |  |  |  |  |  | *Likely* |

| **Variable info in dataset**  Name: SND1  Label: SND1  Rating: 1 to 7  Value:  1.00 = Unlikely  4.00 = Neutral  7.00 = Likely |
| --- |

1. Kebanyakan orang dalam hidup saya, berkerja secara beretika mengikut ajaran Islam setiap hari di tempat kerja.

*Most people in my life work in an ethical manner according to Islamic teaching every day at the workplace.*

| Tidak setuju | 1 | 2 | 3 | 4 (Neutral) | 5 | 6 | 7 | Setuju |
| --- | --- | --- | --- | --- | --- | --- | --- | --- |
| *Disagree* |  |  |  |  |  |  |  | *Agree* |

| **Variable info in dataset**  Name: SND2  Label: SND2  Rating: 1 to 7  Value:  1.00 = Disagree  4.00 = Neutral  7.00 = Agree |
| --- |

1. Kebanyakan rakan sekerja saya, berkerja secara beretika mengikut ajaran Islam, setiap hari di tempat kerja.

*Most of my colleagues work in an ethical manner according to Islamic teaching every day at the workplace.*

| Tidak | 1 | 2 | 3 | 4 (Neutral) | 5 | 6 | 7 | Ya |
| --- | --- | --- | --- | --- | --- | --- | --- | --- |
| *No they do not* |  |  |  |  |  |  |  | *Yes they do* |

| **Variable info in dataset**  Name: SND3  Label: SND3  Rating: 1 to 7  Value:  1.00 = No they do not  4.00 = Neutral  7.00 = Yes they do |
| --- |

## 2.3 Perceived Behavioral Control

## 2.3.1 Perceived Behavioral Control – Autonomy (PBCA)

1. Adalah bergantung pada saya, untuk berkerja secara beretika mengikut ajaran Islam, setiap hari di tempat kerja.

*It is up to me to work in an ethical manner according to Islamic teaching every day at the workplace.*

| Tidak setuju | 1 | 2 | 3 | 4 (Neutral) | 5 | 6 | 7 | Setuju |
| --- | --- | --- | --- | --- | --- | --- | --- | --- |
| *Disagree* |  |  |  |  |  |  |  | *Agree* |

| **Variable info in dataset**  Name: PBCA1  Label: PBCA1  Rating: 1 to 7  Value:  1.00 = Disagree  4.00 = Neutral  7.00 = Agree |
| --- |

1. Adalah diluar kawalan saya untuk berkerja dengan cara yang beretika mengikut ajaran Islam setiap hari di tempat kerja.

*It is beyond my control to work in an ethical manner according to Islamic teaching every day at the workplace.*

| Tidak setuju | 1 | 2 | 3 | 4 (Neutral) | 5 | 6 | 7 | Setuju |
| --- | --- | --- | --- | --- | --- | --- | --- | --- |
| *Disagree* |  |  |  |  |  |  |  | *Agree* |

| **Variable info in dataset**  Name: PBCA2  Label: PBCA2  Rating: 1 to 7  Value (in dataset):  1.00 = Agree  4.00 = Neutral  7.00 = Disagree  Note: This item is a reverse item. In the dataset, it had already been reverse-coded. |
| --- |

1. Sejauh mana anda fikir ianya dibawah kawalan anda, untuk berkerja secara beretika mengikut ajaran Islam, setiap hari di tempat kerja.

*How much control do you think you have to work in an ethical manner according to Islamic teaching every day at the workplace?*

| Diluar kawalan | 1 | 2 | 3 | 4 (Neutral) | 5 | 6 | 7 | Dalam kawalan |
| --- | --- | --- | --- | --- | --- | --- | --- | --- |
| *No control* |  |  |  |  |  |  |  | *Complete control* |

| **Variable info in dataset**  Name: PBCA3  Label: PBCA3  Rating: 1 to 7  Value:  1.00 = No control  4.00 = Neutral  7.00 = Complete control |
| --- |

## 2.3.2 Perceived Behavioral Control – Capacity (PBCC)

1. Saya yakin saya boleh berkerja secara beretika mengikut ajaran Islam, setiap hari di tempat kerja.

*I am confident I can work in an ethical manner according to Islamic teaching every day at the workplace.*

| Salah | 1 | 2 | 3 | 4 (Neutral) | 5 | 6 | 7 | Betul |
| --- | --- | --- | --- | --- | --- | --- | --- | --- |
| *False* |  |  |  |  |  |  |  | *True* |

| **Variable info in dataset**  Name: PBCC1  Label: PBCC1  Rating: 1 to 7  Value:  1.00 = False  4.00 = Neutral  7.00 = True |
| --- |

1. Bagi saya, berkerja dengan cara yang beretika mengikut ajaran Islam setiap hari di tempat kerja, adalah:

*For me to work in an ethical manner according to Islamic teaching every day at the workplace, is:*

| Susah | 1 | 2 | 3 | 4 (Neutral) | 5 | 6 | 7 | Senang |
| --- | --- | --- | --- | --- | --- | --- | --- | --- |
| *Difficult* |  |  |  |  |  |  |  | *Easy* |

| **Variable info in dataset**  Name: PBCC2  Label: PBCC2  Rating: 1 to 7  Value:  1.00 = Difficult  4.00 = Neutral  7.00 = Easy |
| --- |

1. Jika saya inginkannya, saya boleh berkerja secara beretika mengikut ajaran Islam.

*If I want to, I can work in an ethical manner according to Islamic teaching.*

| Tidak setuju | 1 | 2 | 3 | 4 (Neutral) | 5 | 6 | 7 | Setuju |
| --- | --- | --- | --- | --- | --- | --- | --- | --- |
| *Disagree* |  |  |  |  |  |  |  | *Agree* |

| **Variable info in dataset**  Name: PBCC3  Label: PBCC3  Rating: 1 to 7  Value:  1.00 = Disagree  4.00 = Neutral  7.00 = Agree |
| --- |

## 2.4 Behavioral Intention (BI)

1. Saya berniat untuk berkerja secara beretika mengikut ajaran Islam, setiap hari di tempat kerja.

*I intend to work in an ethical manner according to Islamic teaching every day at the workplace.*

| Tidak mungkin | 1 | 2 | 3 | 4 (Neutral) | 5 | 6 | 7 | Mungkin |
| --- | --- | --- | --- | --- | --- | --- | --- | --- |
| *Unlikely* |  |  |  |  |  |  |  | *Likely* |

| **Variable info in dataset**  Name: BI1  Label: BI1  Rating: 1 to 7  Value:  1.00 = Unlikely  4.00 = Neutral  7.00 = Likely |
| --- |

1. Saya bercadang untuk berkerja secara beretika mengikut ajaran Islam, setiap hari di tempat kerja.

*I plan on working in an ethical manner according to Islamic teaching every day at the workplace.*

| Tidak setuju | 1 | 2 | 3 | 4 (Neutral) | 5 | 6 | 7 | Setuju |
| --- | --- | --- | --- | --- | --- | --- | --- | --- |
| *Disagree* |  |  |  |  |  |  |  | *Agree* |

| **Variable info in dataset**  Name: BI2  Label: BI2  Rating: 1 to 7  Value:  1.00 = Disagree  4.00 = Neutral  7.00 = Agree |
| --- |

1. Saya berazam untuk berkerja secara beretika mengikut ajaran Islam, setiap hari di tempat kerja.

*I am determined to work in an ethical manner according to Islamic teaching every day at the workplace.*

| Tidak setuju | 1 | 2 | 3 | 4 (Neutral) | 5 | 6 | 7 | Setuju |
| --- | --- | --- | --- | --- | --- | --- | --- | --- |
| *Disagree* |  |  |  |  |  |  |  | *Agree* |

| **Variable info in dataset**  Name: BI3  Label: BI3  Rating: 1 to 7  Value:  1.00 = Disagree  4.00 = Neutral  7.00 = Agree |
| --- |

1. Saya ingin berkerja secara beretika mengikut ajaran Islam, setiap hari di tempat kerja.

*I want to work in an ethical manner according to Islamic teaching every day at the workplace.*

| Tidak mungkin | 1 | 2 | 3 | 4 (Neutral) | 5 | 6 | 7 | Mungkin |
| --- | --- | --- | --- | --- | --- | --- | --- | --- |
| *Unlikely* |  |  |  |  |  |  |  | *Likely* |

| **Variable info in dataset**  Name: BI4  Label: BI4  Rating: 1 to 7  Value:  1.00 = Unlikely  4.00 = Neutral  7.00 = Likely |
| --- |

1. Saya jangka akan berkerja dengan cara yang beretika mengikut ajaran Islam, setiap hari di tempat kerja.

*I expect to work in an ethical manner according to Islamic teaching every day at the workplace.*

| Tidak setuju | 1 | 2 | 3 | 4 (Neutral) | 5 | 6 | 7 | Setuju |
| --- | --- | --- | --- | --- | --- | --- | --- | --- |
| *Disagree* |  |  |  |  |  |  |  | *Agree* |

| **Variable info in dataset**  Name: BI5  Label: BI5  Rating: 1 to 7  Value:  1.00 = Disagree  4.00 = Neutral  7.00 = Agree |
| --- |

1. Saya akan berusaha untuk berkerja secara beretika mengikut ajaran Islam, setiap hari di tempat kerja.

*I will make an effort to work in an ethical manner according to Islamic teaching every day at the workplace.*

| Saya tidak akan | 1 | 2 | 3 | 4 (Neutral) | 5 | 6 | 7 | Saya akan |
| --- | --- | --- | --- | --- | --- | --- | --- | --- |
| *I definitely will not* |  |  |  |  |  |  |  | *I definitely will* |

| **Variable info in dataset**  Name: BI6  Label: BI6  Rating: 1 to 7  Value:  1.00 = I definitely will not  4.00 = Neutral  7.00 = I definitely will |
| --- |

## 2.5 Retrospective Behavior (RB) of Islamic Ethical Work Behavior (IEWB)

1. Selama ini, saya memang berkerja secara beretika mengikut ajaran Islam, setiap hari di tempat kerja.

*In the past, I have been working in an ethical manner according to Islamic teaching every day at the workplace.*

| Salah | 1 | 2 | 3 | 4 (Neutral) | 5 | 6 | 7 | Betul |
| --- | --- | --- | --- | --- | --- | --- | --- | --- |
| *False* |  |  |  |  |  |  |  | *True* |

| **Variable info in dataset**  Name: IEWB_RB  Label: IEWB - RB  Rating: 1 to 7  Value:  1.00 = False  4.00 = Neutral  7.00 = True |
| --- |

# 3. Islamic Religiosity

Islamic beliefs scale (IBS) with 5 items, Islamic ethical principles scale (IEPS) with 10 items, Islamic universality scale (IUS) with 4 items, Islamic duty scale (IDS) with 5 items, Islamic obligation scale (IOS) with 5 items, Islamic exclusivism scale (IES) with 4 items, and global religiousness scale (GRS) with 2 items.

## Islamic Beliefs Scale (IBS)

1. Saya percaya adanya Tuhan.

*I believe in God.*

1. Saya percaya adanya Hari Akhirat.

*I believe in the Day of Judgement.*

1. Saya percaya pada kewujudan syurga dan neraka.

*I believe in the existence of heaven and hell.*

1. I percaya pada kewujudan malaikat, jin, and syaitan.

*I believe in the existence of the angels, the jinn, and satan.*

1. Saya percaya pada nabi-nabi yang telah diutuskan dan kitab-kitab yang diturunkan.

*I believe in all the prophets that God sent and in the sacred texts that were revealed to them.*

| **Variable info in dataset**  Name: Each item is named as IBS followed by a number, e.g., IBS1.  Label: Each item is labeled similarly, e.g., IBS1.  Rating: Three choices are provided. See Value.  Value:  0.00 = No  1.00 = Uncertain  2.00 = Yes  Note: Boxes were provided in the printed questionnaire next to the items with the appropriate choices stated (see Rating).  Source: Abu-Raiya, H., Pargament, K. I., Mahoney, A., & Stein, C. (2008). A Psychological Measure of Islamic Religiousness: Development and evidence for reliability and validity. The International Journal for the Psychology of Religion, 18(4), 291–315. doi:10.1080/10508610802229270 |
| --- |

## Islamic Ethical Principles Scale (IEPS)

1. Kerana Islam, saya berusaha menjadi seorang yang rendah diri.

*Because of Islam, I strive to be a humble person.*

1. Kerana Islam, saya melakukan yang terbaik untuk memuliakan ibu bapa saya.

*Because of Islam, I do my best to honor my parents.*

1. Kerana Islam, saya menolong sanak-saudara dan jiran tetangga.

*Because of Islam, I try to help my relatives and neighbors.*

1. Kerana Islam, saya menolong orang yang susah dan anak yatim.

*Because of Islam, I try to help the needy and the orphans.*

1. Kerana Islam, saya berusaha menjadi seorang yang bersikap toleransi.

*Because of Islam, I strive to be a tolerant person.*

1. Kerana Islam, saya mengelak dari memakan daging babi.

*Because of Islam, I refrain from eating pork.*

1. Kerana Islam, saya mengelak dari meminum arak.

*Because of Islam, I refrain from drinking alcohol.*

1. Kerana Islam, saya mengelak dari melakukan zina.

*Because of Islam, I refrain from having sex before marriage or outside of marriage.*

1. Kerana Islam, saya tidak memilih bunuh diri sebagai pilihan.

*Because of Islam, I do not consider suicide.*

1. Kerana Islam, saya mengelak dari bergosip.

*Because of Islam, I refrain from backbiting or gossiping.*

| **Variable info in dataset**  Name: Each item is named as IEPS followed by a number, e.g., IEPS1.  Label: Each item is labeled similarly, e.g., IEPS1.  Rating: This scale is rated on a 5-point Likert-type scale: 1 (*strongly disagree*); 2 (*disagree*); 3 (*neutral*); 4 (*agree*); 5 (*strongly* *agree*).  Value: Refer to Rating.  Note: Boxes were provided in the printed questionnaire next to the items with the appropriate choices stated (see Rating).  Source: Abu-Raiya, H., Pargament, K. I., Mahoney, A., & Stein, C. (2008). A Psychological Measure of Islamic Religiousness: Development and evidence for reliability and validity. The International Journal for the Psychology of Religion, 18(4), 291–315. doi:10.1080/10508610802229270 |
| --- |

## Islamic Universality Scale (IUS)

1. Saya menganggap setiap orang Islam di dunia ini sebagai saudara saya.

*I consider every Muslim in the world as my brother or sister.*

1. Saya memahami dan bersimpati akan penderitaan setiap orang Islam di dunia ini.

*I empathize with the suffering of every Muslim in the world.*

1. Salah satu sumber utama rasa bangga diri saya adalah diri saya sebagai seorang Muslim.

*One of my major sources of pride is being a Muslim.*

1. Saya percaya konsep kesaudaraan adalah salah satu dari ajaran Islam.

*I believe brotherhood / sisterhood to be one of the basic tenets of Islam.*

| **Variable info in dataset**  Name: Each item is named as IUS followed by a number, e.g., IUS1.  Label: Each item is labeled similarly, e.g., IUS1.  Rating: This scale is rated on a 5-point Likert-type scale: 1 (*strongly* *disagree*); 2 (*disagree*); 3 (*neutral*); 4 (*agree*); 5 (*strongly* *agree*).  Value: Refer to Rating.  Note: Boxes were provided in the printed questionnaire next to the items with the appropriate choices stated (see Rating).  Source: Abu-Raiya, H., Pargament, K. I., Mahoney, A., & Stein, C. (2008). A Psychological Measure of Islamic Religiousness: Development and evidence for reliability and validity. The International Journal for the Psychology of Religion, 18(4), 291–315. doi:10.1080/10508610802229270 |
| --- |

## Islamic Duty Scale (IDS)

1. Berapa kerap kali anda sembahyang?

*How often do you pray?*

1. Berapa kerap kali anda berpuasa?

*How often do you fast?*

1. Berapa kerapkan anda pergi ke masjid?

*How often do you go to the masjid?*

1. Selain ketika bersembahyang, berapa kerapkah anda membaca Al-Quran atau mendengarnya?

*Except in prayers, how often do your read or listen to the Holy Quran?*

1. Selain ketika bersembahyang, berapa kerapkah anda melakukan zikir atau tasbih?

*Except in prayers, how often do you engage in zikr or tasbih?*

| **Variable info in dataset**  Name: Each item is named as IDS followed by a number, e.g., IDS1.  Label: Each item is labeled similarly, e.g., IDS1.  Rating: Each item has different ratings.   - IDS1: The rating and choices provided are: 0 (*never*); 1 (*a few times a year*); 2 (*several times a month*); 3 (*several times a week*); 4 (*most of the times the 5 daily prayers*); 5 (*five times a day or more*). - IDS2: The rating and choices provided are: 0 (*never*); 1 (*few times in life*); 2 (*few days of the month of Ramadan each year*); 3 (*half of the month of Ramadan each year*); 4 (*the whole month of Ramadan each year*); 5 (*other religious days, or sunnah fasts, in addition to Ramadan*). - IDS3: The rating and choices provided are: 0 (*never*); 1 (*a few times in my life*); 2 (*a few times a year*); 3 (*a few times a month*); 4 (*about once or twice a week*); 5 (*once a day or more*). - IDS4: The rating and choices provided are: 0 (*never*); 1 (*a few times in my life*); 2 (*a few times a year*); 3 (*a few times a month*); 4 (*about once or twice a week*); 5 (*once a day or more*). - IDS5: The rating and choices provided are: 0 (*never*); 1 (*a few times in my life*); 2 (*a few times a year*); 3 (*a few times a month*); 4 (*about once or twice a week*); 5 (*once a day or more*).   Value: See Rating.  Note: Boxes were provided in the printed questionnaire next to the items with the appropriate choices stated (see Rating).  Source: Abu-Raiya, H., Pargament, K. I., Mahoney, A., & Stein, C. (2008). A Psychological Measure of Islamic Religiousness: Development and evidence for reliability and validity. The International Journal for the Psychology of Religion, 18(4), 291–315. doi:10.1080/10508610802229270 |
| --- |

## Islamic Obligation Scale (IOS)

1. Saya berpuasa di bulan Ramadan kerana saya akan berasa kurang senang jika tidak melakukannya.

*I fast in Ramadan because I would feel bad if I did not.*

1. Saya sembahyang kerana jika tidak, Allah tidak akan merestui saya.

*I pray because if I do not, Allah will disapprove of me.*

1. Saya membaca Al-Quran kerana saya akan rasa bersalah jika tidak melakukannya.

*I read the Holy Quran because I would feel guilty if I did not.*

1. Saya pergi ke masjid kerana memang sepatutnya seseorang itu pergi ke masjid.

*I go to the masjid because one is supposed to go to the masjid.*

1. Saya pergi ke masjid kerana orang lain akan mencela saya jika saya tidak melakukannya.

*I go to the masjid because others would disapprove of me if I did not.*

| **Variable info in dataset**  Name: Each item is named as IOS followed by a number, e.g., IOS1.  Label: Each item is labeled similarly, e.g., IOS1.  Rating: This scale is rated on a 4-point Likert-type scale: 1 (*not at all true*); 2 (*usually not true*); 3 (*usually true*); 4 (*very true*). However, this instrument provides the choice of not applicable, with no rating point attached to it, and treated as missing value.  Value: Refer to Rating.  Note: Boxes were provided in the printed questionnaire next to the items with the appropriate choices stated (see Rating).  Source: Abu-Raiya, H., Pargament, K. I., Mahoney, A., & Stein, C. (2008). A Psychological Measure of Islamic Religiousness: Development and evidence for reliability and validity. The International Journal for the Psychology of Religion, 18(4), 291–315. doi:10.1080/10508610802229270 |
| --- |

## Islamic Exclusivism Scale (IES)

1. Islam adalah panduan lengkap dari Allah yang membimbing kepada kebahagiaan dan keselamatan di dunia dan akhirat, yang wajib dipatuhi.

*Islam is Allah’s complete, unfailing guide to happiness and salvation, which must be followed.*

1. Antara semua orang di muka bumi ini, orang Islam mempunyai hubungan istimewa dengan Allah, kerana mereka adalah orang yang paling percaya akan kebenaran yang diturunkan-Nya dan berusaha sedaya-upaya mematuhi hukum-Nya.

*Of all the people on this earth, Muslims have a special relationship with Allah because they believe the most in His revealed truths and try the hardest to follow His laws.*

1. Adalah lebih penting menjadi orang yang baik daripada beriman kepada Allah dan Islam.

*It is more important to be a good person than to believe in Allah and the right religion.*

1. Islam adalah panduan yang terbaik untuk menyembah Allah dan tidak boleh dikompromi.

*Islam is the best way to worship Allah and should never be compromised.*

| **Variable info in dataset**  Name: Each item is named as IES followed by a number, e.g., IES1.  Label: Each item is labeled similarly, e.g., IES1.  Rating: This scale is rated on an 8-point Likert-type scale: -4 (*very strongly disagree*); -3 (*strongly disagree*); -2 (*moderately disagree*); -1 (*slightly disagree*); 1 (*slightly agree*); 2 (*moderately agree*); 3 (*strongly agree*); 4 (*very strongly agree*). However, for IES3, it is a reverse-coded item, which had been reverse-coded in the dataset for standardization with other items.  Value: Refer to Rating.  Note: Boxes were provided in the printed questionnaire next to the items with an empty box for participants to fill with a rating (see Rating).  Source: Abu-Raiya, H., Pargament, K. I., Mahoney, A., & Stein, C. (2008). A Psychological Measure of Islamic Religiousness: Development and evidence for reliability and validity. The International Journal for the Psychology of Religion, 18(4), 291–315. doi:10.1080/10508610802229270 |
| --- |

## Global Religiousness Scale (GRS)

1. Apakah deskripsi anda mengenai pegangan beragama anda?

*How do you describe your religiousness?*

1. Apakah deskripsi anda mengenai spirituality anda?

*How do you describe your spirituality?*

| **Variable info in dataset**  Name: Each item is named as GRS followed by a number, e.g., GRS1.  Label: Each item is labeled similarly, e.g., GRS1.  Rating: This scale is rated on a 5-point Likert-type scale: 1 (*very low*); 2 (*low*); 3 (*average*); 4 (*high*); 5 (*very high*).  Value: Refer to Rating.  Note: Boxes were provided in the printed questionnaire next to the items with the appropriate choices stated (see Rating).  Source: Abu-Raiya, H., Pargament, K. I., Mahoney, A., & Stein, C. (2008). A Psychological Measure of Islamic Religiousness: Development and evidence for reliability and validity. The International Journal for the Psychology of Religion, 18(4), 291–315. doi:10.1080/10508610802229270 |
| --- |

# 4. Composite Variables

All measures are computed to yield their respective mean values as composite variables. For example, all items under ATB are averaged, resulting to ATBMean. This was done for all items. However, there were some missing responses for IOS. Hence, only those without missing responses were computed.
